# Traditional but Not HIV-Related Factors Are Associated with Nonalcoholic Fatty Liver Disease in Asian Patients with HIV-1 Infection

**DOI:** 10.1371/journal.pone.0087596

**Published:** 2014-01-31

**Authors:** Takeshi Nishijima, Hiroyuki Gatanaga, Takuro Shimbo, Hirokazu Komatsu, Yuichi Nozaki, Naoyoshi Nagata, Yoshimi Kikuchi, Mikio Yanase, Shinichi Oka

**Affiliations:** 1 AIDS Clinical Center, Center for Clinical Sciences, National Center for Global Health and Medicine, Tokyo, Japan; 2 Gastroenterology/Hepatology, Center for Clinical Sciences, National Center for Global Health and Medicine, Tokyo, Japan; 3 Department of Clinical Study and Informatics, Center for Clinical Sciences, National Center for Global Health and Medicine, Tokyo, Japan; 4 Center for AIDS Research, Kumamoto University, Kumamoto, Japan; 5 Department of Community Care, Saku Central Hospital, Nagano, Japan; Kaohsiung Medical University Hospital, Kaohsiung Medical University, Taiwan

## Abstract

**Background:**

The prevalence and factors associated with nonalcoholic fatty liver disease (NAFLD) are largely unknown in HIV-1 monoinfected patients.

**Methods:**

The present study elucidated the prevalence and factors associated with NAFLD among Asian patients with HIV-1 infection who underwent abdominal ultrasonography between January 2004 and March 2013. Diagnosis of NAFLD was based on the liver to kidney contrast and diffusion in hepatic echogenicity. Uni- and multi-variate logistic regression analyses were applied to estimate factors associated with NAFLD.

**Results:**

435 Asian patients free of chronic hepatitis B or C virus infection and without excessive alcohol intake were analyzed. NAFLD was diagnosed in 135 (31%) patients. Obesity (BMI >30 kg/m^2^) was evident in 18 (4.1%) patients, and BMI was >25 kg/m^2^ in 103 (24%). Multivariate analysis identified higher BMI (per 1 kg/m^2^ increment, adjusted OR = 1.198; 95% CI, 1.112–1.290; p<0.001), dyslipidemia (adjusted OR = 2.045; 95% CI, 1.183–3.538; p = 0.010), and higher ALT to AST ratio (per 1 increment, adjusted OR = 3.557; 95% CI, 2.129–5.941; p<0.001) as significant factors associated with NAFLD. No HIV-specific variables, including treatment with dideoxynucleoside analogues (didanosine, stavudine, and zalcitabine) and cumulative duration of antiretroviral therapy (ART), were associated with NAFLD.

**Conclusions:**

The incidence of NALFD among Asian patients with HIV-1 infection is similar to that in Western countries. NAFLD was associated with high BMI, dyslipidemia, and high ALT/AST ratio, but not with HIV-related factors. The results highlight the importance of early recognition and management of NAFLD and traditional factors associated with NAFLD for Asian patients with HIV-1 infection.

## Introduction

Nonalcoholic fatty liver disease (NAFLD) is characterized by the presence of fat infiltration in the liver in the absence of excessive alcohol consumption or other causes of liver disease, such as viral hepatitis, and is considered the most common cause of fatty liver [1]. NAFLD is a major health issue since it can lead to fibrosis, cirrhosis, liver cancer, and mortality [2]. Although the prevalence of NAFLD seems increasing in parallel with the current epidemic of obesity, it varies among the general population according to the geographical area; for example, the prevalence of NAFLD in the US ranges from 10 to 46% [3], [4], whereas in Asia it is 5–30% [5].

In the general population, obesity, type 2 diabetes mellitus, dyslipidemia, and metabolic syndrome are established conditions associated with NAFLD [6]. In addition to abovementioned environmental risk factors, genetic factors are also associated with the incidence of NAFLD [7]. However, only two studies (one from Italian metabolic clinic and the other from American naval hospital) have previously examined the prevalence and associated factors with NAFLD in patients infected with HIV-1 only (those without chronic hepatitis C virus (HCV) infection) [8], [9]. At this stage, it is unknown whether variables specific to HIV-1 infection, such as HIV-1 viral load and cumulative years of antiretroviral therapy (ART) are associated with NAFLD. Although the use of so called “D drugs”: dideoxynucleoside analogues [didanosine (ddI), stavudine (d4T), and zalcitabine (ddC)], a subgroup of antiretroviral agents nucleoside reverse transcriptase inhibitors (NRTI), is reported to be associated with NAFLD, others have argued against such relation [8], [9].

Liver diseases are important causes of morbidity and mortality among patients with HIV-1 infection [10]–[12], especially following the wide availability of ART and substantial improvement in prognosis of such patients [13]. Currently, there is no information on the prevalence and associated factors related to NAFLD among patients with HIV-1 infection in Asia, the region with the second largest number of patients with HIV-1 infection. The present study was designed to elucidate the prevalence and associated factors, including D drug use, with NAFLD in Asian patients with HIV-1 infection.

## Methods

### Ethics statement

This study was approved by the Human Research Ethics Committee of the National Center for Global Health and Medicine, Tokyo. Each participant provided a written informed consent for the clinical and laboratory data to be used and published for research purposes. The study was conducted according to the principles expressed in the Declaration of Helsinki.

### Study design

We performed a single-center cross-sectional study of HIV-1-infected patients using the abdominal ultrasonography data and medical records at the National Center for Global Health and Medicine, Tokyo, Japan. Our facility is one of the largest clinics for patients with HIV infection in Japan with approximately 3,500 registered patients [14]. The study population was HIV-infected patients, aged >17 years, who underwent routine abdominal ultrasonography conducted by certified medical technologists at the Physiological Examination Unit of the hospital, between January 1, 2004 and March 31, 2013. The following exclusion criteria were employed in this study; 1) HCV or hepatitis B virus (HBV) infection defined by positive hepatitis C antibody or positive hepatitis B surface antigen, respectively, 2) use of injection drugs, 3) hemophilia, because all HIV-infected hemophiliacs in Japan were exposed to HCV through contaminated blood products [15], and 4) alcohol consumption >20 g of ethanol per day for males and >10 g/day for females. Fatty liver was diagnosed based on hyperechogenicity of the liver compared to renal cortex and diffusion in hepatic echogenicity [8], [16], [17]. The ultrasonographic images and diagnosis were double-checked and confirmed by radiologists, hepatologists, or gastroenterologists. If abdominal ultrasonography was conducted more than once during the study period, the latest data were used for the study.

### Measurements

The potential risk factors for NAFLD were selected according to previous studies and collected from the medical records [18], [19], together with the basic demographic data. These factors included age, sex, race, body weight, body mass index (BMI) = {bodyweight (kg)/[(height (m)]^2^}, and presence or absence of other medical conditions [diabetes mellitus, defined by use of glucose-lowering agents or fasting plasma glucose ≥126 mg/dl or plasma glucose ≥200 mg/dl on two different days, dyslipidemia, defined by current treatment with lipid-lowering agents or two successive measurements of either low-density lipoprotein cholesterol (LDL-C) >140 mg/dl, high-density lipoprotein cholesterol (HDL-C) <40 mg/dl, total cholesterol (TC) >240 mg/dl, triglyceride (TG) >500 mg/dl, and hypertension defined by current treatment with antihypertensive agents or two successive measurements of systolic blood pressure ≥140 mmHg or diastolic blood pressure ≥90 mmHg at the clinic]. Data on smoking status and alcohol consumption were collected through a structured interview conducted at the first visit as part of routine clinical practice by the nurses specializing at the HIV outpatient care. Patients were divided into three groups according to the smoking status: non-smokers, low (<20 cigarettes/day) and heavy smokers (≥20 cigarettes/day). They were also divided according to alcohol consumption into two groups: non-drinkers and light drinkers (<20 g ethanol/day for men and <10 g ethanol/day for women). The values of alanine aminotransferase (ALT), aspartate aminotransferase (AST), TC, LDL-C, HDL-C, and TG within three months and closest to the day ultrasonography was conducted were collected. HIV-specific variables, such as CD4 cell count, HIV viral load, ART-experienced or ART-naïve, ART regimen at ultrasonography, history of AIDS, and duration of ART were also collected. The duration of D drugs use, as a possible risk factor for NAFLD, was collected, regardless of continuation of these drugs at the time of abdominal ultrasonography [9], [20]. Patients were divided into four groups according to duration of treatment with D drugs; no D drugs use, <1 year exposure, 1–3 years of use, and >3 years of use. In our clinic, it is customary for the patient to visit the clinic once a month before the initiation of ART and until the suppression of HIV-1 viral load, but the visit interval is extended up to every three months after viral load suppression.

### Statistical analysis

Baseline characteristics were compared between patients with and without NAFLD, using the Student's *t*-test or χ^2^ test (Fisher's exact test) for continuous or categorical variables, respectively. Univariate logistic regression analysis was used to identify factors associated with NAFLD. Basic demographics, such as age and sex, and variables with *p* values <0.05 in univariate analysis were entered into multivariate logistic regression models. ALT, and TG and LDL-C were not added to the model, based on their multicollinearity with ALT to AST ratio and dyslipidemia, respectively. Statistical significance was defined as two-sided *p* value <0.05. We used the odds ratio (OR) and 95% confidence interval (95%CI) to estimate the association of each variable with NAFLD. All statistical analyses were performed with The Statistical Package for Social Sciences ver. 20.0 (SPSS, Chicago, IL).

## Results

**Figure 1:**
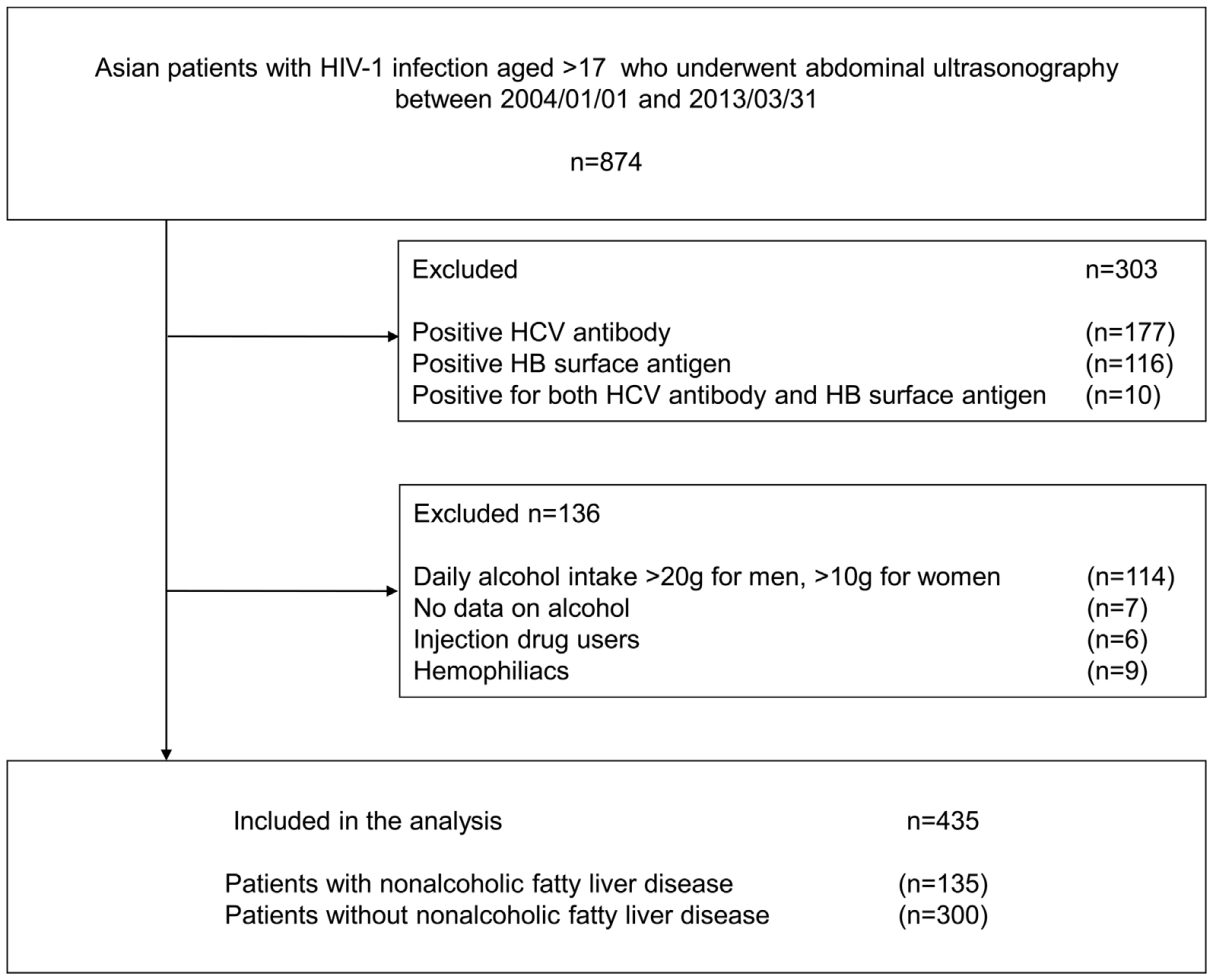
Patient enrollment process.

Of the total of 895 patients with HIV-1 infection who underwent abdominal ultrasonography during the study period, 435 were included in the analysis (Figure 1). NAFLD was diagnosed by abdominal ultrasonography in 135 cases, with a prevalence of 31%. None of these patients had any ultrasonographic finding compatible with cirrhosis. Table 1 shows the characteristics of the study population, patients with NAFLD, and those without NAFLD. The study patients were mostly East Asian males with maintained CD4 count [median 349/µl, interquartile range (IQR) 203–512], and approximately half of the patients had suppressed viral load. Obesity (BMI >30 kg/m^2^) was noted in 18 (4.1%) patients, and BMI was >25 kg/m^2^ in 103 (24%). Body weight was significantly heavier in patients with NAFLD (median 71 kg, IQR 61–78 kg), compared with non-NAFLD (median 61 kg, IQR 55–68 kg, p<0.001), as was BMI (median 25, IQR 21.7–27.5 versus median 21.5, IQR 20–23.3, p<0.001). Dyslipidemia (p<0.001), hypertension (p = 0.019), high ALT (p = 0.017), high LDL-C (p = 0.041), hypertriglyceridemia (p = 0.008), and high CD4 count (p = 0.001) were significantly more common in patients with NAFLD than those without (Table 1). On the other hand, history of D drug use and cumulative years of ART were not significantly different between the two groups.

**Table 1:** Basic demographics of the entire study population, patients with NAFLD and without NAFLD.

	Total (n = 435)	NAFLD (n = 135)	No NAFLD (n = 300)	P^a^
Age (years)¶	40 (35–50)	41 (36–48)	40 (34–55)	0.669
Male sex, n (%)	406 (93)	129 (96)	277 (92)	0.299
Body weight (kg)¶	63 (57–73)	71 (61–78)	61 (55–68)	<0.001
Body mass index, (kg/m^2^)¶	22.1 (20.2–24.9)	25 (21.7–27.5)	21.5 (20–23.3)	<0.001
Body mass index >25 kg/m^2^, n (%)	103 (24)	64 (49)	39 (13)	<0.001
Body mass index >30 kg/m^2^, n (%)	18 (4.1)	16 (12)	2 (1)	<0.001
East Asian origin, n (%)	424 (98)	133 (99)	291 (97)	0.515
Diabetes mellitus, n (%)	22 (5)	9 (7)	13 (4)	0.345
Dyslipidemia, n (%)	120 (28)	55 (41)	65 (22)	<0.001
Hypertension, n (%)	86 (20)	36 (27)	50 (17)	0.019
ALT (IU/l)¶	26 (17–47)	47 (25–80)	22 (16–33)	0.017
AST (IU/l)¶	25 (19–37)	31 (21–50)	23 (18–31)	0.152
ALT to AST ratio¶	1.05 (0.8–1.42)	1.42 (1.02–1.76)	1 (0.74–1.21)	<0.001
Low-density lipoprotein cholesterol (mg/dl)¶	102 (85–126)	111 (90–129)	101 (83–125)	0.041
High-density lipoprotein cholesterol (mg/dl)¶	44 (35–52)	43 (34–52)	44 (35–54)	0.701
Triglyceride (mg/dl)¶	162 (104–233)	189 (125–254)	149 (96–226)	0.008
Total cholesterol (mg/dl)¶	175 (150–205)	179 (151–208)	177 (149–226)	0.202
Smoking status, by no. of cigarettes per day				0.244
None, n (%)	247 (57)	84 (62)	163 (55)	
<20, n (%)	82 (19)	20 (15)	62 (21)	
≥20, n (%)	105 (24)	31 (23)	74 (25)	
Alcohol consumption				1.000
None, n (%)	209 (48)	65 (48)	144 (48)	
Moderate (<20 g/day for men, <10 g/day for women), n (%)	226 (52)	70 (52)	156 (52)	
HIV-specific variables				
CD4 cell count (cells/µL)¶	349 (203–512)	377 (230–591)	338 (172–480)	0.001
HIV load (log_10_ copies/mL)¶	1.70 (1.70–4.45)	1.70 (1.70–4.36)	1.70 (1.70–4.52)	0.508
HIV load <50 copies/mL, n (%)	227 (52)	73 (55)	154 (52)	0.602
Homosexual contact, n (%)	377 (87)	120 (89)	257 (86)	0.446
History of ddI/ddC/d4T exposure, n (%)	128 (29)	37 (27)	91 (30)	0.571
ART duration (years)¶	1.4 (0–5.6)	1.4 (0–6.1)	1.6 (0–5.4)	0.844
Current antiretroviral therapeutic regimen				
Ritonavir-boosted PI plus 2NRTIs, n (%)	186 (43)	58 (43)	128 (43)	1.000
NNRTI plus 2NRTIs, n (%)	44 (10)	14 (10)	30 (10)	1.000
Treatment naïve, n (%)	152 (35)	46 (34)	106 (35)	0.829
History of AIDS, n (%)	156 (36)	51 (38)	105 (35)	0.590

¶ Data are median (interquartile range). Four missing values in variable HIV load<50 copies/mL.

a χ^2^ test or Fisher's exact test was used for categorical data, and Student's *t* test for continuous variables.

NAFLD, nonalcoholic fatty liver disease; ALT, alanine aminotransferase; AST, aspartate aminotransferase; ddI, didanosine; ddC, zalcitabine; d4T, stavudine; ART, antiretroviral therapy; PI, protease inhibitor; NRTI, nucleoside reverse transcriptase inhibitor; NNRTI, non-nucleoside reverse transcriptase inhibitor; AIDS, acquired immunodeficiency syndrome.

Univariate analysis showed a significant association between NAFLD and the following non-HIV specific variables (Table 2): higher BMI (per 1 kg/m^2^ increment, OR = 1.282; 95% CI, 1.197–1.373; p<0.001), dyslipidemia (OR = 2.475; 95% CI, 1.594–3.842; p<0.001), hypertension (OR = 1.818; 95% CI, 1.117–2.961; p = 0.016), ALT to AST ratio (per 1 increment, OR = 4.831; 95% CI, 3.073–7.594; p<0.001), higher ALT (per 10 IU/l increment, OR = 1.027; 95% CI, 1.002–1.053; p = 0.034), higher triglyceride (per 10 mg/dl increment, OR = 1.021; 95% CI, 1.005–1.038; p = 0.010), and higher LDL-C (per 10 mg/dl increment, OR = 1.096; 95% CI, 1.003–1.196; p = 0.042). Among HIV-specific variables, only higher CD4 count was associated with NAFLD (per 1/µl increment, OR = 1.001; 95% CI, 1.001–1.002; p = 0.002)(Table 3). On the other hand, older age (per 1 year increment, OR = 0.996; 95% CI, 0.980–1.013; p = 0.668) and diabetes mellitus (OR = 1.577; 95% CI, 0.657–3.784; p = 0.308) were not associated with NAFLD. Compared to no D drug use, history of D drug use was not associated with NAFLD (Any to <1 year of D drug use, n = 42, OR = 0.956; 95% CI, 0.476–1.919; p = 0.899)(1 to 3 years of D drug use, n = 46, OR = 1.137; 95% CI, 0.592–2.184; p = 0.699)(>3 years of D drug use, n = 40, OR = 0.533; 95% CI, 0.237–1.200; p = 0.129)(Table 3).

**Table 2:** Univariate analysis to estimate the associations of non HIV-specific variables with nonalcoholic fatty liver disease.

	Odds ratio	95%CI	P value
Male sex	1.785	0.710–4.491	0.218
Age per 1 year increment	0.996	0.980–1.013	0.668
Body mass index per 1 kg/m^2^ increment	1.282	1.197–1.373	<0.001
Alcohol consumption			
No drinking	Reference	Reference	Reference
Ethanol <20 g/day for men, <10 g/day for women	0.994	0.662–1.493	0.977
Smoking status			
Non smoker	Reference	Reference	Reference
<20 cigarettes/day	0.626	0.354–1.105	0.106
≥20 cigarettes/day	0.813	0.495–1.334	0.412
Diabetes mellitus	1.577	0.657–3.784	0.308
Dyslipidemia	2.475	1.594–3.842	<0.001
Hypertension	1.818	1.117–2.961	0.016
ALT to AST ratio per 1 increment	4.831	3.073–7.594	<0.001
ALT per 10 IU/l increment	1.027	1.002–1.053	0.034
AST per 10 IU/l increment	1.034	0.986–1.084	0.169
Triglyceride per 10 mg/dl increment	1.021	1.005–1.038	0.010
Low-density lipoprotein cholesterol per 10 mg/dl increment	1.096	1.003–1.196	0.042
Total cholesterol per 10 mg/dl increment	1.037	0.981–1.096	0.202
High-density lipoprotein cholesterol per 10 mg/dl increment	1.032	0.878–1.215	0.700

CI, confidence interval; ALT, alanine aminotransferase; AST, aspartate aminotransferase.

**Table 3:** Univariate analysis to estimate the association of HIV-specific variables with nonalcoholic fatty liver disease.

	Odds ratio	95%CI	P value
ddI/ddC/d4T use	0.867	0.552–1.362	0.536
No ddI/ddC/d4T use (n = 307)	Reference	Reference	
<1 year of ddI/ddC/d4T use (n = 42)	0.956	0.476–1.919	0.899
1–3 years of ddI/ddC/d4T use (n = 46)	1.137	0.592–2.184	0.699
>3 years of ddI/ddC/d4T use (n = 40)	0.533	0.237–1.200	0.129
ART exposure			
Treatment naïve (n = 152)	Reference	Reference	
<2 years of ART exposure (n = 80)	1.110	0.620–1.985	0.726
2–6 years of ART exposure n = 100)	0.941	0.541–1.637	0.830
>6 years of ART exposure (n = 103)	1.135	0.664–1.943	0.643
CD4 count per 1/µl increment	1.001	1.001–1.002	0.002
HIV viral load per log_10_/ml increment	0.955	0.833–1.094	0.507
HIV viral load <50 copies/ml	1.138	0.755–1.715	0.538
History of AIDS	1.128	0.740–1.718	0.576
Treatment naive	0.946	0.617–1.450	0.799

OR, odds ratio; CI, confidence interval; ddI, didanosine; ddC, zalcitabine; d4T, stavudine; ART, antiretroviral therapy; AIDS, acquired immunodeficiency syndrome.

Among patients treated with D drugs (n = 128), the median time period since withdrawal was 3.46 years (IQR 1.03–6.29). Compared to treatment-naïve patients, ART use was not associated with NAFLD as well (<2 year of ART exposure, n = 80, OR = 1.110; 95% CI, 0.620–1.985; p = 0.726) (2 to 6 years of ART exposure, n = 100, OR = 0.941; 95% CI, 0.541–1.637; p = 0.830) (>6 year of ART exposure, n = 103, OR = 1.135; 95% CI, 0.664–1.943; p = 0.643)(Table 3).

Multivariate analyses identified the following variables as independently associated with NAFLD: BMI (per 1 kg/m^2^ increment, adjusted OR = 1.198; 95% CI, 1.112–1.290; p<0.001), dyslipidemia (adjusted OR = 2.045; 95% CI, 1.183–3.538; p = 0.010), ALT to AST ratio (per 1 increment, adjusted OR = 3.557; 95% CI, 2.129–5.941; p<0.001)(Table 4).

**Table 4:** Multivariate analysis of independent variables associated with nonalcoholic fatty liver disease (n = 408).

	Adjusted OR	95%CI	P value
Male sex	1.953	0.640–5.966	0.240
Age 1 year increment	1.005	0.983–1.027	0.672
Body mass index per 1 kg/m^2^ increment	1.198	1.112–1.290	<0.001
Dyslipidemia	2.045	1.183–3.538	0.010
ALT to AST ratio per 1 increment	3.557	2.129–5.941	<0.001
Hypertension	0.959	0.510–1.805	0.897
CD4 count per 1/µl increment	1.001	0.999–1.002	0.336

OR, odds ratio; CI, confidence interval; ALT, alanine aminotransferase; AST, aspartate aminotransferase.

## Discussion

To our knowledge, this is the first study that investigated the prevalence and associated factors of NAFLD in Asian patients with HIV-1 infection, and is the largest study that focused on NAFLD in patients with HIV-1 monoinfection (without chronic hepatitis C infection). The prevalence of NAFLD in this study was 31%, which is comparable to 31% at the Naval hospital in San Diego, US, and 36.9% at the metabolic clinic in Modena, Italy [8], [9]. Multivariate analysis indicated that traditional predictors for NAFLD in the general population, such as higher BMI, dyslipidemia, and ALT to AST ratio [6], were significantly associated with NAFLD, whereas HIV-specific variables, including history of D drug use and cumulative years of ART, were not associated with NAFLD.

Our result of nearly one third of Asian patients with HIV-1 monoinfection have NAFLD highlights the importance of screening for NAFLD among this patient population, due to the potential progression of NAFLD to liver fibrosis, cirrhosis, and liver cancer [2], [21]. In addition, the finding that higher BMI, dyslipidemia, and ALT to AST ratio were associated with NAFLD warrants aggressive approach to life-style changes and keeping optimal body weight, as well as the management of dyslipidemia. This is particularly important because the metabolic syndrome, obesity, type 2 diabetes mellitus, and dyslipidemia are widely prevalent and are increasing among the general population in Asia [5]. Our study identified obesity in 4.1% of the study population (BMI >30 kg/m^2^), the number that is similar to that reported from the Italian metabolic clinic (4.9%), although much lower than that reported in US (14.8%) [8], [9]. Our results showed that the prevalence of NAFLD in Asian patients with HIV-1 infection is as high as that reported in the above two studies, and warrants the need for paying attention to this disease in Asian patients with HIV-1 infection.

Interestingly, the present study did not identify HIV-specific variables, especially treatment with D drugs, to be associated with NAFLD. D drugs (dideoxynucleoside analogues; ddI, d4T, and ddC), a subgroup of NRTIs, inhibit mitochondrial DNA (mDNA) polymerase γ, resulting in depletion of mDNA in the liver [22], and causes mitochondria toxicity with potential fatal lactic acidosis and hepatic steatosis [23]–[25]. However, previous studies on patients with HIV monoinfection (without chronic hepatitis C infection) showed conflicting results with regard to the relation between NAFLD and D drug use [8], [9]. The present study also did not find significant association between D drug use and NAFLD. Considering that D drugs are rarely used in resource-rich settings and their use is also rapidly decreasing in resource-limited settings, especially after 2010 revision of WHO guidelines, which eliminated d4T from the first line therapy (http://whqlibdoc.who.int/publications/2010/9789241599764_eng.pdf), it is probably plausible to say that more focus needs to be put on traditional predictors for NAFLD, such as obesity and dyslipidemia, rather than D drug use when screening and managing NAFLD in patients with HIV-1 infection.

There are several limitations to our study. First, the diagnosis of NAFLD was achieved by use of ultrasonography, although histological confirmation of NAFLD by liver biopsy is considered the gold standard [16]. Because it is also difficult to grade the severity of fat infiltration in the liver by ultrasonography, the present study could not distinguish nonalcoholic steatohepatitis (NASH), the more severe form of NAFLD [6], [16], [19]. However, liver biopsy is an invasive and costly procedure. Compared to histopathology and other imaging devices, such as computed tomography (CT) and magnetic resonance imaging (MRI), the reliability and accuracy of ultrasonography in the diagnosis of fatty liver has been well-established [16]. Other advantages of ultrasonography includes low cost, safety, and availability, compared with liver biopsy, CT, and MRI [16]. Second, because the study population comprised of mostly males, the results of the present study might not apply to female patients. Third, we cannot exclude possible overestimation of the prevalence of NAFLD in this study since the study population included patients who underwent abdominal ultrasonography in clinical practice. However, considering that the two previous reports on NAFLD in HIV-monoinfected patients included only patients with dyslipidemia and hyperglycemia at the metabolic clinic [9], and almost exclusively military personnel at the naval hospital [8], respectively, the present study confers clinically useful information derived from routine clinical practice with comparatively unrestricted patient population at a large urban HIV clinic.

In conclusion, the present study demonstrated that the prevalence of NAFLD in Asian patients with HIV-1 infection was 31%, which is comparable to the studies from Western Europe and US. NAFLD was significantly associated with traditional predictors for NAFLD, such as higher BMI, dyslipidemia, and ALT to AST ratio, but not with any HIV-specific variable, including history of D drug use and cumulative years of ART. The results highlight the importance of early recognition and management of NAFLD and its traditional predictors, in order to prevent further progression of NAFLD in Asian patients with HIV-1 infection.
